# Total RNA sequencing reveals multilevel microbial community changes and functional responses to wood ash application in agricultural and forest soil

**DOI:** 10.1093/femsec/fiaa016

**Published:** 2020-02-03

**Authors:** Toke Bang-Andreasen, Muhammad Zohaib Anwar, Anders Lanzén, Rasmus Kjøller, Regin Rønn, Flemming Ekelund, Carsten Suhr Jacobsen

**Affiliations:** 1 Department of Environmental Science, Aarhus University, RISØ Campus, Roskilde, 4000, Denmark; 2 Department of Biology, University of Copenhagen, DK-2100, Copenhagen, Denmark; 3 Department of Conservation of Natural Resources, NEIKER-Tecnalia, Bizkaia Technology Park, E-48160, Derio, Spain; 4 AZTI-Tecnalia, Herrera Kaia, E-20110, Pasaia, Spain; 5 Ikerbasque, Basque Foundation for Science, E-48013, Bilbao, Spain; 6 Arctic Station, University of Copenhagen, 3953, Qeqertarsuaq, Greenland

**Keywords:** metatranscriptomics, total RNA, wood ash, biodiversity, soil biota, protozoa

## Abstract

Recycling of wood ash from energy production may counteract soil acidification and return essential nutrients to soils. However, wood ash amendment affects soil physicochemical parameters that control composition and functional expression of the soil microbial community. Here, we applied total RNA sequencing to simultaneously assess the impact of wood ash amendment on the active soil microbial communities and the expression of functional genes from all microbial taxa. Wood ash significantly affected the taxonomic (rRNA) as well as functional (mRNA) profiles of both agricultural and forest soil. Increase in pH, electrical conductivity, dissolved organic carbon and phosphate were the most important physicochemical drivers for the observed changes. Wood ash amendment increased the relative abundance of the copiotrophic groups Chitinonophagaceae (Bacteroidetes) and Rhizobiales (Alphaproteobacteria) and resulted in higher expression of genes involved in metabolism and cell growth. Finally, total RNA sequencing allowed us to show that some groups of bacterial feeding protozoa increased concomitantly to the enhanced bacterial growth, which shows their pivotal role in the regulation of bacterial abundance in soil.

## INTRODUCTION

Wood ash from energy production is often considered a waste product (Vance 1996; Demeyer, Voundi Nkana and Verloo 2001) despite that recycling of wood ash may have beneficial effects as it counteracts acidification and returns essential nutrients to soil (Demeyer, Voundi Nkana and Verloo 2001; Augusto, Bakker and Meredieu 2008). Wood combustion is becoming more popular in several countries and increased reuse of wood ash as soil amendment holds the potential to improve the sustainability of this practice (Karltun *et al*. 2008; Huotari *et al*. 2015). However, wood ash application affects several soil physicochemical parameters important to the structure and function of microbial communities, e.g. pH, electrical conductivity and dissolved organic carbon (DOC; Ohno and Susan Erich 1990; Demeyer, Voundi Nkana and Verloo 2001; Pitman 2006; Augusto, Bakker and Meredieu 2008; Hansen *et al*. 2017; Maresca, Hyks and Astrup 2017). As the soil microbiota carries out an array of key biochemical processes (Blagodatskaya and Kuzyakov 2013), knowledge of its response to disturbance is important, not least in production soils due to potential impact on soil fertility.

The soil microbiome, which includes prokaryotes as well as microeukaryotes, is one of the most diverse and complex biomes on Earth. It has a pivotal role in nutrient cycling and carbon sequestration and is a key component in the maintenance of soil fertility of managed ecosystems (Wall *et al*. 2012; Fierer 2017). Wood ash amendment causes changes in soil microbiome composition, activity and quantity (Perkiömäki and Fritze 2002; Aronsson and Ekelund 2004; Huotari *et al*. 2015). Ash amendment induces changes in community structure followed by increased microbial activity and growth, which is usually explained by the increased soil pH brought about by the alkaline oxides in the ash (Cruz-Paredes *et al*. 2017; Vestergård *et al*. 2018). Still, some studies show no or only minor microbial response to wood ash application (Aronsson and Ekelund 2004; Huotari *et al*. 2015).

Only few studies have concomitantly analyzed microorganisms from all domains of life (i.e. Archaea, Bacteria and Eukaryotes) and most of these rely on cultivation, model organisms or molecular fingerprinting, which provide only limited resolution of taxonomical and functional responses. Total RNA sequencing, or metatranscriptomics, makes it possible to investigate active soil microbial communities from all domains of life, including their transcriptional activity, simultaneously. By targeting RNA—and not DNA—most of the biases associated with relic DNA are avoided. Relic DNA can, because of its relative slow degradation in soil, result in biases with delayed functional and community responses (Carini *et al*. 2016). Total RNA sequencing allows for the study of immediate regulatory responses to environmental changes (Carvalhais *et al*. 2012), and it has proven useful in the assessment of active microbial communities’ functional roles in soil (Urich *et al*. 2008; Epelde *et al*. 2015; Geisen *et al*. 2015; Hultman *et al*. 2015; Schostag *et al*. 2019).

We therefore aimed to investigate how the active soil prokaryotic and microeukaryotic communities in agricultural and forest soil responded structurally and functionally (transcriptional) to wood ash application. Both soil types are relevant for large-scale application of wood ash. We applied wood ash in concentrations corresponding to field application of 0, 3, 12 and 90 t ha^−1^, where 3 t ha^−1^ is the currently allowed dose in Scandinavian countries. We expected wood ash to increase soil pH, electrical conductivity and DOC and therefore hypothesized that (i) the pH increase would favor bacteria more than fungi, (ii) the nutrients in the wood ash would benefit the copiotrophic microbial groups, (iii) multitrophic responses would appear gradually over time after wood ash application and (iv) microbial stress responses would be observable in the transcriptome.

## MATERIALS AND METHODS

### Soils and wood ash

We used two contrasting soils for the experiment. The first was a loamy sand (Typic Hapludult) from the plough layer (0–10 cm) of an agricultural field (Research Center Foulum, DK; 56°29′42′N 9°33′36′E). The other was from the O-horizon (0–10 cm) of a forest (Gedhus, DK; 56°16′38′N 09°05′12′E). The forest is a second-generation Norway spruce stand [*Picea abies* (L.) Karst.] on Podzol heathland. Qin *et al*. (2017) provide soil characteristics for both soils. On both sites, we removed visible plant parts before taking ten 100 g soil samples within a 30 m^2^ area. The 10 samples from each site were sieved (4 mm), pooled and stored in the dark for 14 days at 4°C until further processing.

Wood ash was a mixture of bottom and fly ash from a heating plant (Brande, Denmark) produced by combustion of wood chips from predominantly coniferous trees. We homogenized the ash by sieving (2 mm). Maresca, Hyks and Astrup (2017) provide a list of mineral nutrients and heavy metals in the ash.

### Microcosm setup and incubation

We prepared microcosms in triplicates of 50 g soil in 250 ml sterilized airtight glass jars. We mixed the ash thoroughly with soil-to-ash concentrations corresponding to field application of 0, 3, 12 and 90 t ash ha^−1^. The water content was adjusted to 50% of the water holding capacity of the two soils. We prepared 12 microcosms for each soil-ash combination to allow four destructive samplings, i.e. a total of 96 microcosms. Samples were also collected at the start of the experiment. Microcosms were incubated at 10°C in the dark and all microcosms were opened once a week inside an LAF bench to maintain aerobic conditions.

### Physicochemical soil parameters

At destructive sampling, after 3, 10, 30 and 100 days of incubation, we prepared soil extracts from 15 g soil and 75 ml sterile ddH_2_O followed by 1 h shaking and settling for 0.5 h. In the supernatant, we measured electrical conductivity using a TetraCon 325 electrode adapted to a conductivity meter Cond 340i (WTW, Weilheim, Germany) and pH using a pH electrode (Sentix Mic, WTW, Weilheim, Germany) connected to pH meter Multi 9310 (WTW, Weilheim, Germany). The remaining supernatant was filtered (5C filters; Advantec, Tokyo, Japan; 1 μm pore size) and analyzed for DOC, nitrate (NO_3_^−^), ammonium (NH_4_^+^) and phosphate (PO_4_^3−^). DOC concentrations were determined on a TOC-5000A (Shimadzu, Kyoto, Japan). Nitrate, ammonium and phosphate concentrations were determined by flow injection analysis (FIAstar 5000, FOSS, Hillerød, Denmark), following the manufacturer's instructions.

### Nucleic acid extraction, qPCR and library preparation for sequencing

RNA and DNA were co-extracted from 2 g soil samples using the RNA PowerSoil Total RNA Isolation Kit (MOBIO, Carlsbad, CA, USA) in combination with DNA Elution Accessory Kit (MOBIO), following the manufacturer's protocol. The soil for nucleic acid extraction were immediately frozen in liquid nitrogen (N) after collection (to preserve RNA) and subsequently stored at −80°C until extraction. Agricultural soil amended with the highest ash concentration had an RNA yield below detection limit and was not sequenced.

We quantified 16S rRNA and ITS2 gene copies (DNA level) using qPCR. 16S rRNA genes were amplified in technical duplicates using a CFX Connect (Bio-Rad, Richmond, VA, USA). We used a dilution series of genomic DNA from *Escherichia coli* K-12 (with seven copies of 16S rRNA genes) as a standard (Blattner *et al*. 1997). The master mix consisted of 2 µl bovine serum albumin (BSA; 20 mg/ml; BIORON, Ludwigshafen, Germany), 10 µl SsoFast EvaGreen Supermix (Bio-Rad), 0.8 μl of primer 341f (5′-CCTAYGGGRBGCASCAG-3′), 0.8 μl of primer 806r (5′-GGACTACNNGGGTATCTAAT-3′; Hansen *et al*. 2012), 1 μl of 10× diluted template and 5.4 µl of sterile DEPC-treated water. PCR conditions for 16S rRNA gene amplification were 98°C for 15 min, followed by 35 cycles of 98°C for 30 s, 56°C for 30 s and 72°C for 30 s (with fluorescence measurements) and ending with 72°C for 7 min and production of melt curves. The PCR efficiencies for the 16S assays were 96.1 ± 1.0% (SEM, *n* = 3) with *R*^2^ = 0.99 ± 0.001. ITS gene copies were quantified as described for the 16S rRNA above with minor modifications: Vector cloned ITS2 DNA regions from *Aureobasidium pullulans* were included as standards, primers used were gITS7 (5′-GTGARTCATCGARTCTTTG-3′; Ihrmark *et al*. 2012) and ITS4 (5′-TCCTCCGCTTATTGATATGC-3′; White *et al*. 1990), annealing temperature was 60°C and 40 amplification cycles were used. The PCR efficiencies for the ITS assays were 106.0 ± 4.6% with *R*^2^ = 0.99 ± 0.003.

Prior to total RNA library building, we removed potential DNA carryovers using the DNase Max Kit (MOBIO), following the manufacturer's protocol. Successful DNA removal of RNA extracts was tested with the 16S qPCR protocol described above but with 50 amplification cycles: All DNase-treated RNA extracts had Cq values higher than or equal to those of the negative samples (sterile DEPC-treated water as template) and DNA was thereby not present.

Quality of the DNase-treated RNA was tested using RNA 6000 Nano Kit (Agilent, Santa Clara, CA, USA) on a 2100 Bioanalyzer System (Agilent), following the manufacturer's protocol [average RIN number was 7.85 ± 0.13 (SEM, *n* = 69)].

Subsequently, DNase-treated RNA extracts from time points 0, 3, 30 and 100 days were fragmented into ∼150 bp segments and prepared for sequencing using the NEBNext Ultra Directional RNA Library Prep Kit for Illumina in combination with the NEBNext Multiplex Oligos for Illumina (New England BioLabs, Ipswich, MA, USA), according to the manufacturer's protocol. We sequenced the resulting metatranscriptome libraries using HiSeq 2500 (Illumina Inc., San Diego, CA, USA) in high-output mode (8 HiSeq lanes, 125 bp, paired-end reads) at the National High-throughput DNA Sequencing Centre (Copenhagen, Denmark).

### Bioinformatic processing

We obtained a total of 3.3 billion paired sequences (SRA accession number: PRJNA512608) and processed them through the following bioinformatic pipeline (see Table S1, Supporting Information, for numbers of sequences and contigs during the processing steps). Adapters, poly-A tails, sequences shorter than 60 nt and nucleotides with Phred score below 20 at the 5′ and 3′ end of sequences were removed using Cutadapt v.1.9.1 (Martin 2011). Five samples were removed prior to subsequent processing due to low quality of reads (one replicate of 3 t ha^−1^, day 100 from the agricultural soil; two replicates of 0 t ha^−1^, day 0; and two replicates of 0 t ha^−1^, day 100 from the forest soil). Sequences were then sorted into small subunit (SSU) rRNA, large subunit (LSU) rRNA and non-rRNA sequences using SortMeRNA v.2.1 (Kopylova, Noé and Touzet 2012).

#### rRNA

A subset of 1.5 million randomly chosen SSU rRNA sequences per sample was assembled into longer SSU rRNA contigs using EMIRGE (Miller *et al*. 2011). The subset of sequences was done partly to normalize the number of sequences per sample (to deal with unequal sequencing depth, i.e. different numbers of sequences per sample after HiSeq sequencing), partly due to computational constraints. Contigs were taxonomically classified using CREST (Lanzén *et al*. 2012) and rRNA reads were mapped to resulting EMIRGE contigs using BWA (Li and Durbin 2009), as in Epelde *et al*. (2015), resulting in a table of taxonomically annotated read abundance across samples (Datasheet S1, Supporting Information).

#### mRNA

A combined pool of non-ribosomal sequences from all samples was assembled using trinity v.2.0.6 (Grabherr *et al*. 2011). From the resulting assembled contigs, non-coding RNA contigs were filtered away by aligning contigs to the Rfam database v.12.0 (Nawrocki *et al*. 2015) using cmsearch v.1.1.1 with a significant e-value threshold of <10^−3^. Input sequences used for non-ribosomal RNA assembly were then mapped to coding mRNA contigs. We normalized the contigs by removing those with relative expression lower than 1 out of the number of sequences in the dataset with least number of sequences. EMBOSS (Rice, Longden and Bleasby 2000) was used to search six possible open reading frames (ORFs) of the contigs. SWORD (Vaser, Pavlović and Šikić 2016) was used to align ORFs against the Md5nr protein database (Wilke *et al*. 2012). The output was then parsed with custom Python scripts and filtered hits with minimum e-value of 10^−5^ as threshold. Best hit for each contig was then selected based on alignment statistics and annotated against the eggnog hierarchical database v.4.5 (Jensen *et al*. 2008). The output was an abundance table of numbers of sequences assigned to groups of different functional genes (COGs; Datasheet S2, Supporting Information). The mRNA processing has been validated by Anwar *et al*. (2019).

### Statistical analysis and data processing

Statistical validation for both taxonomy and functional abundance was done in R v.3.4.0 (R Core Team 2015) using *vegan* (Oksanen *et al*. 2008). The rRNA abundance was converted into relative abundance and collapsed taxonomically into Archaea, Bacteria and Eukaryota. We further grouped Eukaryota into Fungi, Metazoa, and protists (with main focus on bacterivorous protozoa). We calculated Richness (number of rRNA contigs) and Shannon diversity on the total number of rRNA contigs and abundance of sequence reads mapped to them. Non-metric multidimensional scaling (NMDS) was carried out using Bray–Curtis dissimilarities of community composition (rRNA contigs and abundance of sequence reads mapped to them) between samples. Soil physicochemical parameters were fitted to the resulting NMDS using the function *envfit*. Variables explaining overall differences in community composition were evaluated using the function *Adonis*, which performs permutational analysis of variance (PERMANOVA; 10 000 permutations) using Bray–Curtis dissimilarities as response variable. A forward selection strategy was carried out to only include explanatory variables with significant *P*-values in *Adonis* models.

Significant effects of wood ash amendment and incubation time on taxonomic groups were determined using non-parametric Kruskal–Wallis tests (due to the non-normal distribution of taxon abundances). To separate the pronounced changes in community responses observed at the 90 t ha^−1^ amendment in the forest soil from the less pronounced changes observed at 0–12 t ha^−1^, we performed Kruskal–Wallis tests with wood ash concentration as independent variable for both the ranges of 0–12 and 0–90 t ha^−1^. We also used Kruskal–Wallis to test the effect of time on differential abundances of taxa within the wood ash concentrations separately. *P*-values were adjusted for false discovery rate (FDR) using the Benjamini–Hochberg method in all tests.

NMDS on Bray–Curtis dissimilarities of functional gene compositions (mRNA) and *Adonis* testing were carried out as described above. Likewise were soil physicochemical parameters fitted to the resulting NMDS, as described above. mRNA gene counts between samples were normalized using the DESeq2 algorithm (Love, Huber and Anders 2014). Significantly differentially expressed genes (mRNA) were analyzed using the DESeq2 module of SARTools (Varet *et al*. 2016). These analyses were conducted by pairwise comparisons of gene transcription (mRNA) levels between samples of increasing wood ash concentration to control samples (0 t ha^−1^) at different incubation times. For the forest samples at time 100 days, only one replicate remained for the 0 t ha^−1^ treatment. Therefore, we compared instead the 12 and 90 t ha^−1^ to the 3 t ha^−1^.

We used linear Pearson regression to test for significant correlations between wood ash concentration and time against measured physicochemical parameters. Additionally, we performed two-way ANOVAs with Tukey's post-hoc tests using wood ash concentration and time as explanatory variables, with all physicochemical parameters as dependent variables. Variance homogeneity was tested using Levene's test and normal distribution of data was tested using the Shapiro–Wilk test in combination with QQ-plots prior to ANOVA tests.

We used a significance level of 0.05, unless otherwise explicitly mentioned, and the Results section provide descriptions at this significance level.

## RESULTS

### Physicochemical parameters

Soil pH, electrical conductivity and DOC correlated positively with wood ash concentration for both soils (Table 1). For the 90 t ha^−1^ ash amendment, soil pH increased from 6.4 to 11.5 in the agricultural and from 4.1 to 8.5 in the forest soil (Figure S1, Supporting Information). Similarly, the 90 t ha^−1^ resulted in 15- and 19-fold increases in electrical conductivity for the agricultural and forest soil, respectively. In the agricultural soil, ammonium increased with time in samples both with and without ash amendment, while nitrate showed no significant changes. In the forest soil, ammonium and nitrate increased after 3 days in the 90 t ha^−1^ amendment, followed by a decrease after 30 days. In the other treatments, increased concentrations were observed during the entire incubation period. In both soils, concentrations of dissolved phosphate increased up to 12 t ash ha^−1^ followed by a decrease at 90 t ha^−1^.

**Table 1.** Pearson correlation values (*r*) and associated significance levels between ash dose (field equivalents 0, 3, 12 and 90 t ha^−1^) and incubation time, and soil physicochemical parameters.

	Agricultural soil		Forest soil	
Explanatory variable	Ash dose (t ha^−1^)	Time (days)	Ash dose (t ha^−1^)	Time (days)
pH	0.76***	0.15	0.98***	0.07
Conductivity (µS cm^−1^)	0.82***	0.14	0.99***	0.07
DOC (mg g^−1^ DW soil)	0.74***	0.33*	0.91***	0.05
Ammonium (µg g^−1^ DW soil)	0.05	0.57***	0.40**	0.36
Nitrate (µg g^−1^ DW soil)	−0.45***	0.28*	0.63***	−0.15
Phosphate (µg g^−1^ DW soil)	−0.61*	−0.07	0.26	−0.04

*P< 0.05, **P< 0.01, ***P< 0.001.

### Quantitative PCR

**Figure 1.**
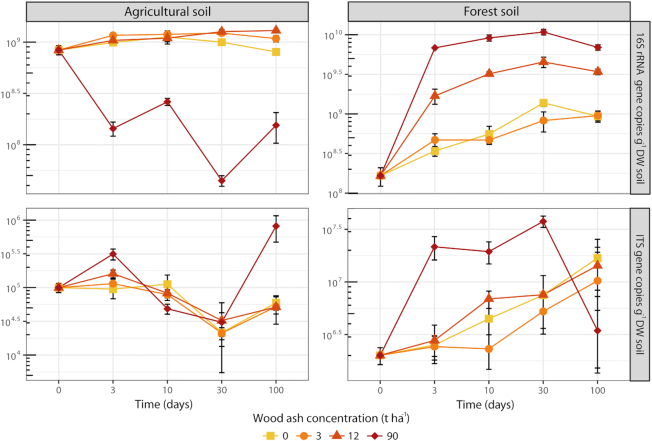
Numbers of 16S rRNA gene copies (top row) and ITS gene copies (bottom row) g^−1^ DW of the agricultural soil (left panel) and the forest soil (right panel) across wood ash concentrations and incubation times. Symbols represent averages with SEM (*n* = 3). The presented data are results from qPCR on DNA. Note logarithmic *y*-axes and different ranges of values on *y*-axes.

Prokaryotic abundance (number of 16S rRNA gene copies) increased in the agricultural soil after the wood ash application of 12 t ha^−1^, but decreased after application of 90 t ha^−1^ (Fig. 1). Fungal abundance (number of ITS copies) remained fairly unchanged over time regardless of ash application with the exception of an increase after 100 days at 90 t ha^−1^. In the forest soil, prokaryotic abundance increased over time for all treatments (Fig. 1); however, addition of 12 and 90 t ha^−1^ resulted in a stronger increase. The fungal abundance in the forest soil showed higher abundance for most of the period with wood ash concentrations of 90 t ha^−1^.

### rRNA—community composition

**Figure 2.**
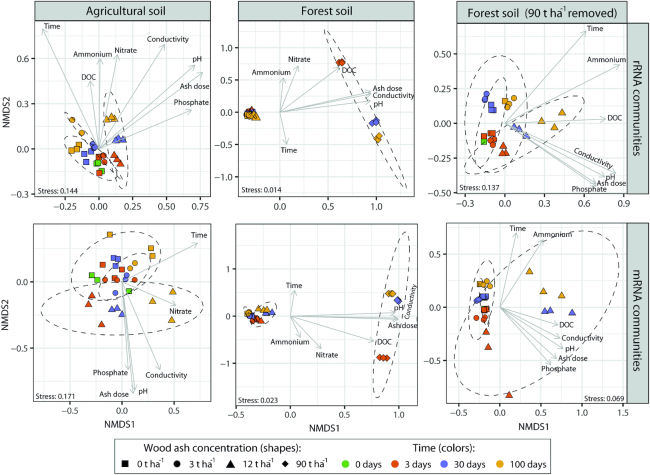
NMDS plots based on Bray–Curtis dissimilarities of the taxonomic (rRNA; top row) and functional (mRNA; bottom row) profiles of an agricultural soil and a forest soil amended with wood ash. Dashed lines represent 95% confidence ellipses around samples with same wood ash concentration. Arrows indicate the direction of fitted physicochemical parameters (using *envfit* function; only significant parameters shown) onto the NMDS ordination space (longer arrows indicate better fit). To improve the resolution of the forest soil at wood ash concentrations 0–12 t ha^−1^, we removed the 90 t ha^−1^ samples and repeated the analysis (rightmost two panels).

The number of unique rRNA contigs ranged from 1216 to 5931 per sample and originated from all domains of life. Community composition differed significantly (*P* < 0.001; *R*^2^ = 0.86; *Adonis*) between the two soil types. For forest soil, amendment with 90 t ha^−1^ resulted in highly altered community composition (Fig. 2) compared to 0–12 t ha^−1^. Though less pronounced, changes from 0–3 to 12 t ha^−1^ were also clearly visible for both soil types (Fig. 2). Moreover, microcosms for particular soil type/ash dose combinations were clearly separated by sampling times (Fig. 2).

In both soils, wood ash dose, incubation time, pH and electrical conductivity correlated to the transformed NMDS community space (Fig. 2). Optimized *Adonis* models (Table 2) supported that wood ash concentration, time, pH and electrical conductivity together significantly explained the variation in microbial communities after ash application in both soils. Additionally, dissolved phosphate significantly explained the variation in microbial communities in both soils up to 12 t ha^−1^ ash amendments and DOC, ammonium and nitrate in the forest soil.

**Table 2.** Explanatory strength of physicochemical variables on rRNA and mRNA dissimilarity profiles of the two soils after ash amendment testing using permutational multivariate analysis of variance (*Adonis*).

	rRNA			mRNA		
Explanatory variable	Agriculture (0–12 t ha^−1^)	Forest (0–90 t ha^−1^)	Forest (0–12 t ha^−1^)	Agriculture (0–12 t ha^−1^)	Forest (0–90 t ha^−1^)	Forest (0–12 t ha^−1^)
pH	0.184***	0.536***	0.216***	0.079*	0.386***	0.224***
Conductivity (µS cm^−1^)	0.081***	0.056***	0.108***	0.140*	0.061***	0.100***
Wood ash concentration (t ha^−1^)	0.113***	0.044***	0.041*	0.063*	0.049***	0.051**
Time (days)	0.089***	0.068***	0.173***	0.092*	0.086***	0.258***
Phosphate (µg g^−1^ DW soil)	0.039*	NS	0.076***	0.065*	NS	0.118***
DOC (mg g^−1^ DW soil)	NS	0.094***	0.038*	NS	0.162***	0.033**
Ammonium (µg g^−1^ DW soil)	NS	0.034**	0.029*	NS	0.066***	0.040**
Nitrate (µg g^−1^ DW soil)	NS	0.027**	0.036*	NS	0.038***	0.028*
Wood ash concentration:time	0.064***	0.015*	0.043*	NS	0.025**	0.039**
Residuals (unexplained variance)	0.430	0.127	0.239	0.560	0.126	0.109

Values refer to *R*^2^ values of the *Adonis* test on Bray–Curtis dissimilarities between samples.

Asterisks refers to significance level (*0.01 < *P*< 0.05, **0.001 < *P*< 0.01, ***P < 0.001).

Non-significant (*P*> 0.05) parameters are written as 'NS'.

### rRNA—taxonomic distribution and diversity

**Figure 3.**
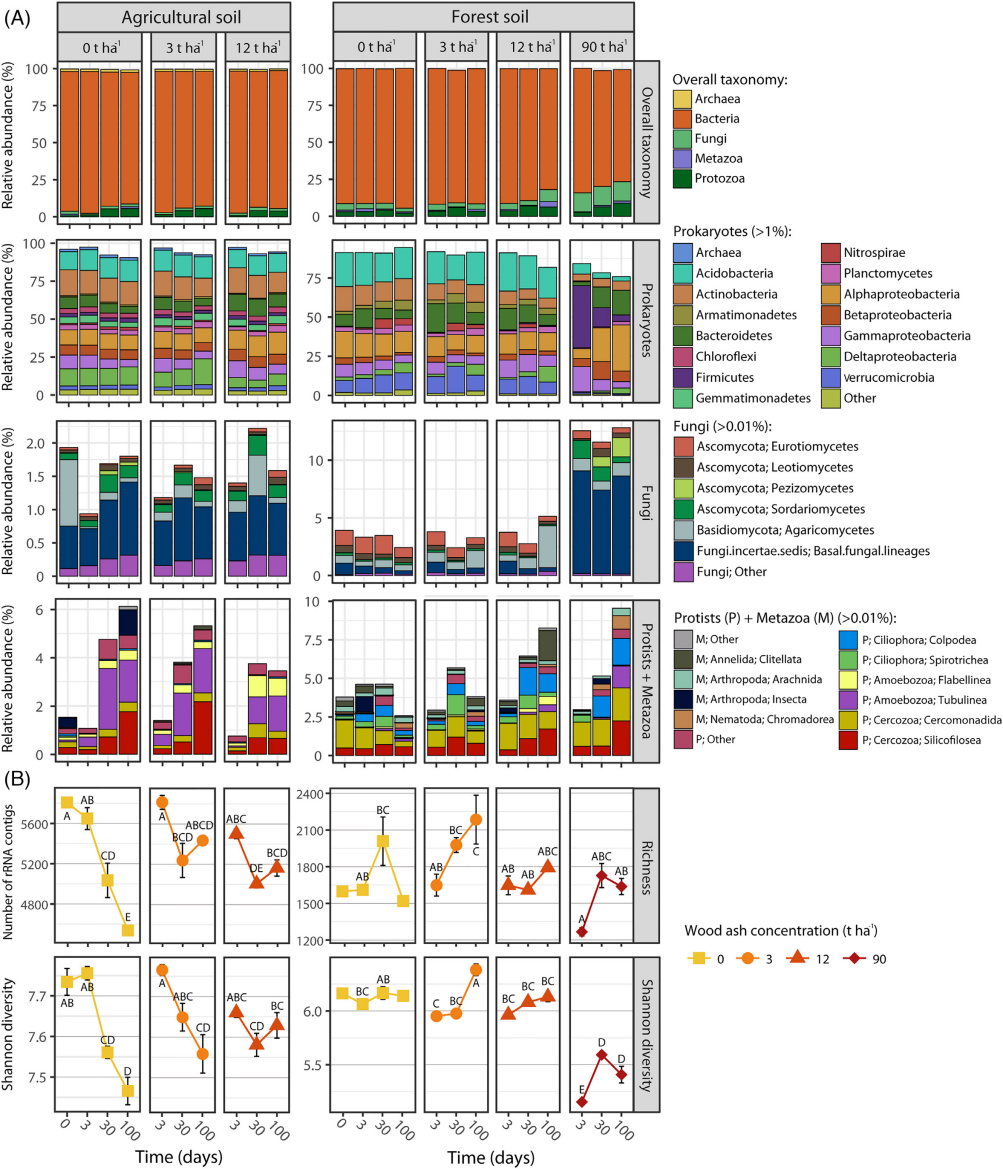
Community composition and diversity across the two soils at increasing wood ash amendment and incubation times based on PCR-free, total RNA-seq. **(A)** The most abundant taxonomic groups (cutoff levels of average relative abundances are shown in legend header) are presented in upper panel (overall taxonomy), i.e. Archaea, Bacteria, Fungi, Protists and Metazoa. Bars represent averages of triplicates [excluding agricultural soil 3 t ha^−1^ at 100 days (*n* = 2), forest soil 0 t ha^−1^ at 0 days (*n* = 1) and forest soil 0 t ha^−1^ at 100 days (*n* = 1)]. **(B)** Richness and Shannon diversity. Statistically significant different richness and diversity measures (*P* < 0.05) between samples within each measure and soil are indicated by different letters. Symbols represent averages, as described for the bar plots.

A majority (85%) of rRNA sequence reads, mapped to rRNA contigs, could be annotated to order rank (99% to phylum and 97% to class rank; Fig. 3A). Fewer sequences could be assigned lower taxonomic ranks (60 and 27% to family and genus level, respectively). Therefore, to include sufficient community information at the lowest taxonomic rank possible, we evaluated possible significant differences in abundance of taxa at order rank as the lowest taxonomic rank (see Datasheets S3 and S4, Supporting Information, for *P*-values and averages of relative abundances, respectively). Furthermore, for taxonomic groups that significantly changed in relative abundance, we examined the community data at lower taxonomic ranks (family and genus) to determine if specific groups were the main contributors for the observed response, as described below. Richness and Shannon diversity decreased in the unamended agricultural soil over time, while ash amendments of 3 and 12 t ha^−1^ counteracted this decrease (Fig. 3B). In the forest soil, these measures generally remained unchanged up to 12 t ha^−1^ amendments (with a single exception of increased richness at 3 t ha^−1^ after 100 days of incubation), while the 90 t ha^−1^ amendment caused reduction of Shannon diversity.

#### Prokaryotic community

In both soil types, the relative abundance of Chitinophagaceae (Bacteroidetes) increased with wood ash application (Fig. 3A). In the agricultural soil, ash amendment also caused increases in Alphaproteobacteria and Betaproteobacteria. In the forest soil, the 3 and 12 t ha^−1^ ash amendments increased Myxococcales (Deltaproteobacteria), while Acidimicrobiia (Actinobacteria) decreased.

In the forest soil, the 90 t ha^−1^ ash amendment resulted in major prokaryotic community changes. Actinobacteria, Acidobacteria, Armatimonadetes and Verrucomicrobia decreased, with Acidobateria having the strongest decrease with an initial relative abundance of 21.7% with no ash amendment to 6.7% 3 days after the 90 t ha^−1^ ash amendment. On the contrary, Firmicutes, Bacteroidetes and Proteobacteria increased after the 90 t ha^−1^ ash amendment. Firmicutes dominated after 3 days, with *Paenibacillus* as most abundant with relative abundance of 21.3%, followed by a gradual decrease toward 1.1% after 100 days. Similarly, Gammaproteobacteria decreased during incubation after an initial increase. Chitinophagaceae (Bacteroidetes) and Rhizobiales (Alphaproteobacteria) showed the opposite temporal trend after 90 t ha^−1^ ash amendment and were most abundant after 100 days; Chitinophagaceae increased in relative abundance from 0.9% after 3 days to 9.8% after 100 days and Rhizobiales increased from 2.5% after 3 days to 16.7% after 100 days.

#### Fungal community

The 3 and 12 t ha^−1^ ash amendments did not affect fungal community composition in the agricultural soil (Fig. 3A). In the forest soil, no major changes were found at low amendments, while application of 90 t ha^−1^ resulted in increase in members of the genus *Mortierella* (incertae sedis) that 3 days after ash amendment became the dominant fungal group with a relative abundance of the total community of 6.5%. Also, the order Hypocreales (Sordariomycetes) and the genus *Peziza* (Pezizomycetes) increased with the 90 t ha^−1^ ash amendment.

#### Microeukaryotic community

In the agricultural soil, the relative abundances of Tubulinea (Amoebozoa), Thaumatomonadida (Cercozoa) and Silicofilosea (Cercozoa) increased over time in all treatments (Fig. 3A). In the forest soil, members belonging to the genus *Colpoda* (Ciliophora) increased with time in all treatments, though more pronouncedly at higher wood ash amendments. Further, Tubulinea (Amoebozoa), Heteromitidae (Cercozoa) and Silicofilosea (Cercozoa) increased in the 12 and 90 t ha^−1^ amendments.

### mRNA—functional genes

A total of 0.9 million sequences were mapped to 463 mRNA contigs. The two soils possessed distinct pools of expressed genes (*P* < 0.001; *R*^2^ = 0.82; *Adonis*; Figure S2 and Datasheet S2, Supporting Information). Accordingly, scaling of Bray–Curtis dissimilarities of mRNA pools between samples on NMDS plots revealed clear clustering patterns and the Bray–Curtis dissimilarities and fitting of physicochemical parameters to these revealed similar trends as for rRNA taxonomic communities (Fig. 2 and Table 2).

**Figure 4.**
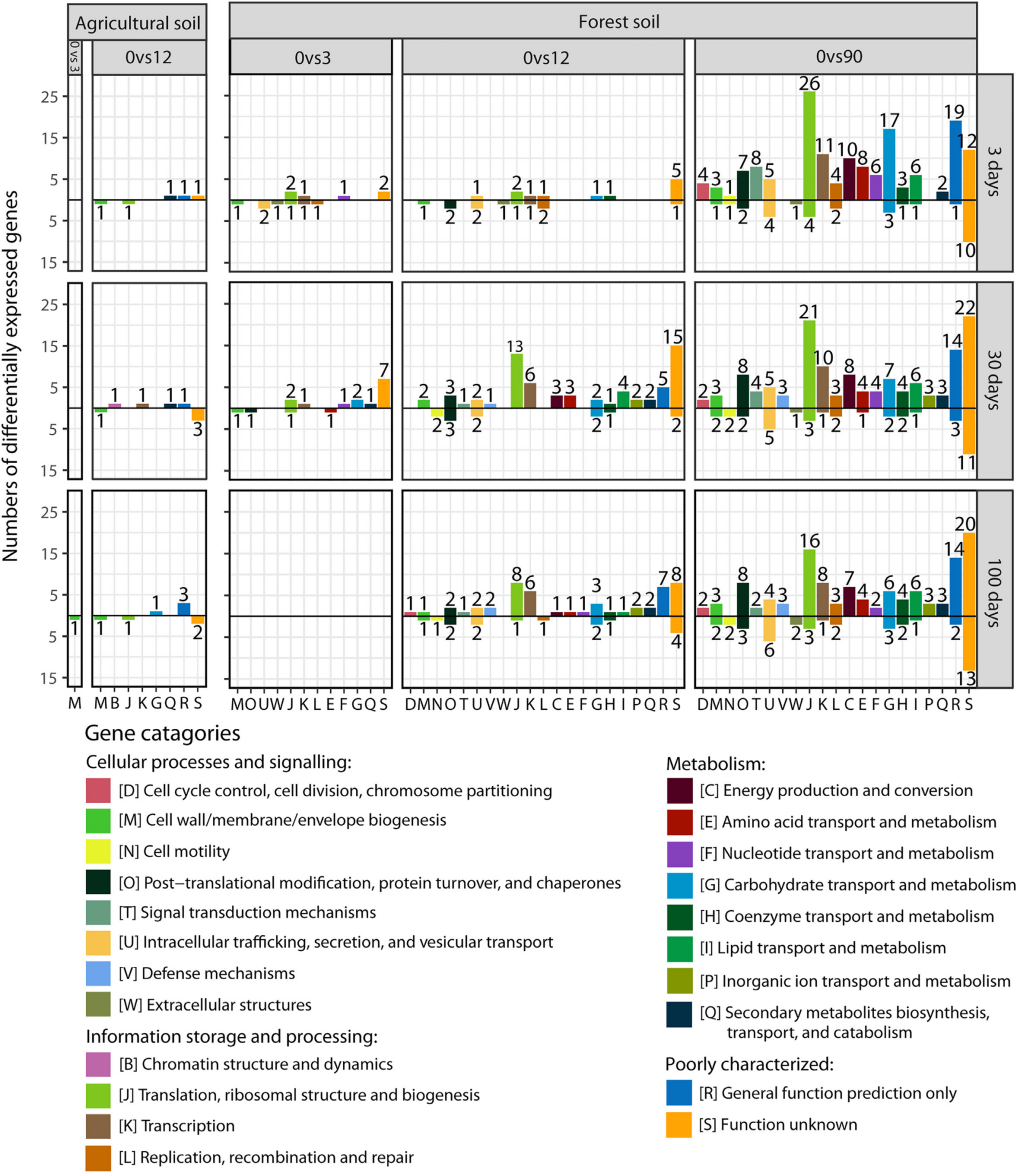
Numbers of differentially expressed genes within functional categories across agricultural and forest soil by pairwise comparisons of gene transcription levels between samples of increasing wood ash concentration to reference samples without ash amendment at different incubation times. ‘0vs3, ‘0vs12’ and ‘0vs90’ denote the wood ash doses compared, i.e. wood ash dose 0 t ha^−1^ compared to 3 t ha^−1^ is written as ‘0vs3’. Increasing and decreasing gene transcription levels are presented above and below the black horizontal zero line, respectively. The pairwise comparisons for forest soil, 100 days, were carried out using 3 t ha^−1^, 100 days, as reference samples because only one replicate was acquired from the 0 t ha^−1^, 100 days, samples (hence the empty plot in 0vs3, 100 days, forest plot). Digits above/below bars represent the number of differentially expressed genes within a gene category.

In the agricultural soil, we observed only minor functional gene responses to time and ash amendment, while more genes were differentially expressed in the forest soil (Fig. 4; see full list of differential expressed genes in Datasheet S5, Supporting Information). Overall, the number of differential expressed functional genes increased with higher wood ash amendments and the ash amendments resulted in more functional genes being upregulated than downregulated. Of the well characterized genes, four functional categories contained most of the differentially expressed genes, i.e. ‘Post-translation modification, protein turnover and chaperones’; ‘Transcription’; ‘Replication, recombination and repair’; and ‘Carbohydrate transport and metabolism’. Furthermore, genes related to stress responses such as chaperones (e.g. ‘COG0443 Molecular Chaperone’), sporulation (e.g. ‘NOG08151 Stage III sporulation protein D’), transmembrane transporters (e.g. ‘COG1744 ABC-type transport system’) and general stress response genes (e.g. ‘COG1825 Ribosomal protein L25–general stress protein Ctc’) increased mainly in the forest soil at 90 t ha^−1^ ash amendments (Figure S3, Supporting Information).

## DISCUSSION

Here, we present the first detailed analysis of changes in soil microbial prokaryotic and eukaryotic communities after amendment with ash using the total RNA sequencing procedure.

### Bacterial responses to wood ash application

The general copiotrophic groups of bacteria, i.e. Bacteroidetes, Alphaproteobacteria and Betaproteobacteria, were stimulated by wood ash application. Members of Bacteroidetes benefit from wood ash application (Noyce *et al*. 2016; Bang-Andreasen *et al*. 2017); they are initial metabolizers of labile carbon and respond positively to increased soil pH and electrical conductivity (Fierer, Bradford and Jackson 2007; Lauber *et al*. 2009; Kim *et al*. 2016). Alpha- and Betaproteobacteria are also generally copiotrophic (Cleveland *et al*. 2007; Fierer, Bradford and Jackson 2007) and Betaproteobacteria thrive in soils with higher pH (Kim *et al*. 2016), whereas Alphaproteobacteria are favored at high N availability (Nemergut *et al*. 2010; Fierer *et al*. 2012).

Acidobacteria and Verrucomicrobia declined after the 90 t ha^−1^ amendment to the forest soil. These phyla are considered oligotrophic (Fierer, Bradford and Jackson 2007; Bergmann *et al*. 2011; Ramirez, Craine and Fierer 2012; Cederlund *et al*. 2014; Kielak *et al*. 2016) and Acidobacteria are generally most abundant under acidic conditions (Rousk *et al*. 2010; Kielak *et al*. 2016). Likewise are many members of the class Acidimicrobiia acidophilic that likely explain the observed decrease in this group after wood ash amendment—the acidophilic members are probably not able to cope with the wood ash-induced increases in soil pH (Johnson *et al*. 2009; Itoh *et al*. 2011). Thus, increases in pH, bioavailable DOC and nutrients induced by wood ash allow copiotrophic groups to thrive at the expense of oligotrophic groups. The shift toward a more copiotrophic-dominated community after ash amendment was further supported by the mRNA profile of the soil. Here, an increasing number of functional genes involved in metabolism and cell growth (‘Translation’, ‘Transcription’ and ‘Replication’) showed significant higher transcription levels. Moreover, the observed increase in 16S rRNA gene copies, as analyzed by qPCR, supports a shift toward a more copiotrophic community with higher average 16S rRNA gene number per genome as well as increased prokaryotic growth (Klappenbach, Dunbar and Schmidt 2000; Roller, Stoddard and Schmidt 2016).

Of the Bacteroidetes, Chitinonophagaceae showed the strongest positive response to wood ash application. Members of this family can degrade a broad spectrum of carbon compounds (Kämpfer *et al*. 2006; Hanada *et al*. 2014). Thus, they are well suited for the ash-induced increased DOC availability. Rhizobiales dominated the increasing Alphaproteobacterial fraction of the forest soil after ash amendment. They are copiotrophs (Starke *et al*. 2016; Lladó and Baldrian 2017) and can degrade organic pollutants and cope with heavy metals (Teng *et al*. 2015). They probably have advantageous properties, as the wood ash induces increase of heavy metals and nutrients in the soils. Deltaproteobacterial Myxococcales responded positively to wood ash amendment in the forest soil. Myxococcales are a group with known fungal-like behaviors including the production of extracellular enzymes involved in carbon degradation and ability to produce spores when nutrients are scarce (Sozinova *et al*. 2005). These traits might give Myxococcales an advantage after wood ash application. Noteworthy, the increase in Myxococcales occurred late in the incubation where especially Chitinophagaceae and Alphaproteobacteria decreased. Myxococcales are ‘micropredators’ and attack and lyse other bacteria, which might explain the increased dominance of this group at the expense of other bacterial groups (Reichenbach 1999).

The increase in 16S rRNA gene copy numbers after ash amendment (up to 12 and 90 t ha^−1^ for the agricultural and forest soil, respectively) is consistent with other reports of increasing bacterial numbers after wood ash application (Bååth and Arnebrant 1994; Fritze *et al*. 2000; Perkiömäki and Fritze 2002; Bang-Andreasen *et al*. 2017; Vestergård *et al*. 2018). The large increase in the forest soil is further consistent with the increased pH as most bacteria thrive better at pH around 7 (Rousk, Brookes and Bååth 2009). Increased prokaryotic growth and a shift toward a copiotrophic-dominated community with higher average 16S rRNA gene number per genome, as described above, are likely causing the 16S rRNA gene copy increase.

The 90 t ha^−1^ ash amendment to the forest soil caused immediate dominance of Firmicutes and Gammaproteobacteria. Both groups are copiotrophs that thrive upon addition of easily degradable carbon and nitrogen to soil, which probably partly explain their success upon ash application (Cleveland *et al*. 2007; Fierer, Bradford and Jackson 2007; Nemergut *et al*. 2010; Fierer *et al*. 2012; Ramirez, Craine and Fierer 2012). However, bacteria from these phyla are also known to be tolerant to heavy metals (Jacquiod *et al*. 2017). Moreover, within Firmicutes the endospore-forming genus *Paenibacillus* dominated (de Hoon, Eichenberger and Vitkup 2010), and we found increased transcription of genes involved in sporulation in these samples. Combined, these capabilities probably enable members of these groups to withstand the initial wood ash-induced changes to the soil, including increased heavy metal concentrations, thereby allowing them to be initial utilizers of newly available labile resources. Reduced diversity at this ash dose further indicates that less organisms can cope with the ash-induced changes to the soil system

### Fungal responses to wood ash application

In both soil types, fungal response to ash amendment was slight compared to the prokaryotic response. Likewise, Högberg, Högberg and Myrold (2007), Rousk, Brookes and Bååth (2009, 2011) and Cruz-Paredes *et al*. (2017) found bacteria to be more stimulated by nutrient addition and increases in pH than fungi. Similarly, effects of ash amendment have been reported by Mahmood *et al*. (2003) and Noyce *et al*. (2016). The 90 t ha^−1^ amendment in the forest soil caused increased ITS gene copy numbers and a fungal community shift with increased dominance of *Mortierella*, *Peziza* and Hypocreales. These fungi are opportunistic saprotrophs with high growth rates and can exploit readily available nutrients before other fungi arrive (Carlile, Watkinson and Gooday 2001; Tedersoo *et al*. 2006; Druzhinina, Shelest and Kubicek 2012). Further, some *Peziza* spp. are early post-fire colonizers adapted to ash conditions (Egger 1986; Rincón *et al*. 2014). The increase in these groups further supports that copiotrophic-like lifestyles are favored by wood ash application.

### Microeukaryote responses to wood ash application

The microeukaryotes also responded to wood ash application in the forest soil, probably because the stimulation of copiotrophic bacteria and fungi provided more food for nematodes and protozoa (Rønn, Vestergård and Ekelund 2012). Ciliates (*Colpoda*), amoebae (Tubulinea) and small heterotrophic flagellates (Heteromitidae and Silicofilosea) increased with more pronounced responses at the later incubation times. Protozoa generally have longer generation times than prokaryotes, and thus need longer time to increase in population size. Further, they cannot start growth before a reasonable bacterial population has been formed (Fenchel 1987; Ekelund, Frederiksen and Rønn 2002). The protozoan increase may explain the small decrease in prokaryotic 16S rRNA gene copies at day 100, where we observed the largest fraction of protozoa. The positively responding protozoa were likely primarily bacterivorous (Ekelund and Rønn 1994; Ekelund 1998), consistent with the decreasing relative fraction of bacterial rRNA sequences and the increasing relative fraction of fungal and protozoan rRNA sequences in the later incubation times after the application of 12 and 90 t ha^−1^ ash. Thus, preferential protozoan grazing on bacteria can explain the relative larger rRNA fraction of fungi and protozoa at day 100. We found no significant effect of ash amendment on microeukaryotes in the agricultural soil, which is consistent with the relative minor effects on prokaryotes and fungi in this soil.

### Stress responses at high wood ash amendments

We recorded increased transcription of stress-response genes at the 90 t ha^−1^ amendments, which supports that this high dose exerts harmful effects on many members of the microbiome. For example, we found increased transcription of genes involved in sporulation. Sporulation is a known survival mechanism to unfavorable conditions (de Hoon, Eichenberger and Vitkup 2010). Also, transmembrane transporter proteins balance osmotic pressure of cells, regulate cytosolic pH and can export toxins such as metals from the cell (Alberts *et al*. 2002; Ma, Jacobsen and Giedroc 2009; Wilkens 2015). Increased activity of transmembrane transporters is probably a response to wood ash-induced osmotic changes to the soil system, increased pH, metal concentration and other toxic compounds. Moreover, chaperones ensure correct folding of proteins and are involved in cellular coping with stress-induced denaturation of proteins (Feder and Hofmann 1999) and the observed increase in transcription level of these probably is a stress response.

### The changes in the microbial communities are linked to physicochemical soil parameters

We found that ash amendment strongly increased soil pH, which is a strong driver of microbial community composition and functioning (Fierer and Jackson 2006; Rousk *et al*. 2010) also after wood ash application (Frostegård *et al*. 1993; Zimmermann and Frey 2002; Högberg, Högberg and Myrold 2007; Peltoniemi *et al*. 2016; Bang-Andreasen *et al*. 2017). DOC and phosphate concomitantly increased. Several factors may contribute to this: (i) pH dependent changes in solubility (Evans *et al*. 2012; Maresca, Hyks and Astrup 2017), (ii) release from dead organisms incapable of coping with the wood ash or wood ash-induced changes to the soil system, (iii) increased mineralization rates after wood ash application (Bååth and Arnebrant 1994; Vestergård *et al*. 2018) and (iv) the phosphorus in the bio-ash (Pitman 2006; Maresca, Hyks and Astrup 2017).

Since pH, conductivity, DOC and phosphate all correlated positively to wood ash concentrations, it is difficult to disentangle the direct effect of these components as they might all be covariates of the wood ash amendments. pH changes induce a cascade of effects in soil parameters and therefore affect mineral nutrient availability, salinity, metal solubility and organic C (Lauber *et al*. 2009). Many of the wood ash-induced changes were likely caused directly or indirectly by the pH increase, which is probably the major reason that pH is an essential driver of taxonomic and functional soil characteristics (Lauber *et al*. 2009; Rousk *et al*. 2010; Fierer 2017; Vestergård *et al*. 2018).

Wood ash contains virtually no nitrogen; hence, measurable effects on soil nitrate and ammonium are probably caused by pH effects on microbial N mineralization (Vestergård *et al*. 2018) and ion solubility (Pitman 2006). Changes in nitrate and ammonium were significant as explanatory variables on the observed rRNA and mRNA dissimilarity profiles of the forest soil but not in the agricultural soil. Forest soil is generally more N limited than agricultural soil, where N is kept at a high level through fertilization.

### Conclusions

We used detailed total RNA sequencing to demonstrate drastic taxonomic and functional changes in the active prokaryotic and eukaryotic microbiomes of agricultural and forest soil after wood ash amendment. Our analyses suggested that increase in pH, electrical conductivity, DOC and phosphate were the main drivers of the observed changes. Wood ash amendment of 3 and 12 t ha^−1^ resulted in increased prokaryotic abundance and dominance of copiotrophic groups and elevated expression of genes involved in metabolism and cell growth. Amendment of 90 t ha^−1^ caused collapse of the microbiome in the agricultural soil, while in the forest soil the copiotrophic microbiome, also including fast-growing saprotrophic fungi, was further stimulated. However, diversity was reduced, and expression of stress response genes increased. Bacterivorous protozoan groups increased as a response to enhanced bacterial growth, which supports that the protozoa have a pivotal role in controlling bacterial abundance in soil following wood ash application. Overall, prokaryotic community and quantity responded more pronouncedly to wood ash amendment than fungi in both forest and agricultural soil.

## Supplementary Material

fiaa016_Supplemental_Files
