# Supplementary material for: Total RNA sequencing reveals multilevel microbial community changes and functional responses to wood ash application in agricultural and forest soil
Source: FEMS Microbiol Ecol. 2020 Feb 3;96(3):fiaa016. doi: 10.1093/femsec/fiaa016 (PMC7028008; doi:10.1093/femsec/fiaa016)
Supplement: fiaa016_Supplemental_Files [file fiaa016_supplemental_files.zip › Bang_Andreasen_et_al_Supplementary_material_TBA.docx]

*Supplementary material for*

**Total RNA-sequencing reveals multi-level microbial community changes and functional responses to wood ash application in agricultural and forest soil**

Toke Bang-Andreasen^1,2^, Muhammad Zohaib Anwar^1^, Anders Lanzén^3,4,5^, Rasmus Kjøller^2^, Regin Rønn^2,6,7^, Flemming Ekelund^2^, and Carsten Suhr Jacobsen^1^.

^1^Department of Environmental Science, Aarhus University, RISØ campus, Roskilde, Denmark. ^2^Department of Biology, University of Copenhagen, Copenhagen, Denmark. ^3^Department of Conservation of Natural Resources, NEIKER-Tecnalia, Bizkaia Technology Park, Derio, Spain. ^4^AZTI-Tecnalia, Herrera Kaia, Pasaia, Spain. ^5^IKERBASQUE, Basque Foundation for Science, Bilbao, Spain. ^6^Key Laboratory of Urban Environment and Health, Institute of Urban Environment, Chinese Academy of Sciences, Xiamen, China. ^7^Arctic Station, University of Copenhagen, Qeqertarsuaq, Greenland.

**Supplementary Table 1 (part 1/2):** Sequence stats during bioinformatic processing

|  |  |  |  |  |  | 1. Quality filtering and sorting into rRNA and mRNA | | | | | | | | |  | | | 2. rRNA processing | | | | | | |  |
| --- | --- | --- | --- | --- | --- | --- | --- | --- | --- | --- | --- | --- | --- | --- | --- | --- | --- | --- | --- | --- | --- | --- | --- | --- | --- |
| Samples | | | |  |  | 1. HiSeq output |  | 1.2. Cutadapt | |  | | 1.3. SortMeRNA | | | |  | | | 2.1 EMIRGE assembly^1^ | | |  | | 2.2 BWA mapping^3^ | |
| Site | Time  (days) | Wood ash (t ha^-1^) | N |  |  | # Raw sequences |  | # Sequences after QC |  | | # SSU rRNA sequences | | # Unaligned (mRNA) sequences |  | | | Assembly stats^2^ (complete contig pool) | | |  |  | | # Sequences mapped to contigs^4^ | |  |
| Agriculture | 0 | 0 | 3 |  |  | 67,827,670 |  | 64,417,976 |  | | 24,720,374 | | 3,043,788 |  | | | # contigs | | | 210,239 |  | | 3,733,602[17,463] | |  |
| Agriculture | 3 | 0 | 3 |  |  | 97,744,520 |  | 92,325,552 |  | | 36,093,938 | | 4,595,954 |  | | | # contigs (>= 0 bp) | | | 210,239 |  | | 3,760,196[16,988] | |  |
| Agriculture | 3 | 3 | 3 |  |  | 79,475,042 |  | 74,431,190 |  | | 29,086,048 | | 3,348,616 |  | | | # contigs (>= 1000 bp) | | | 210,239 |  | | 3,792,021[17,489] | |  |
| Agriculture | 3 | 12 | 3 |  |  | 81,838,422 |  | 77,266,440 |  | | 30,275,752 | | 2,997,240 |  | | | # contigs (>= 5000 bp) | | | 0 |  | | 3,758,900[16,538] | |  |
| Agriculture | 30 | 0 | 3 |  |  | 140,570,504 |  | 132,784,120 |  | | 52,597,386 | | 8,406,872 |  | | | # contigs (>= 10000 bp) | | | 0 |  | | 3,506,699[15,142] | |  |
| Agriculture | 30 | 3 | 3 |  |  | 49,953,868 |  | 40,903,282 |  | | 16,311,690 | | 2,269,774 |  | | | Largest contig | | | 1,943 |  | | 3,620,144[15,756] | |  |
| Agriculture | 30 | 12 | 3 |  |  | 66,856,992 |  | 59,674,826 |  | | 23,340,932 | | 3,102,498 |  | | | Total length | | | 318,179,120 |  | | 3,614,148[15,042] | |  |
| Agriculture | 100 | 0 | 3 |  |  | 96,488,474 |  | 91,851,912 |  | | 36,098,960 | | 5,899,912 |  | | | Total length (>= 0 bp) | | | 318,179,120 |  | | 3,464,616[13,645] | |  |
| Agriculture | 100 | 3 | 2 |  |  | 42,609,030 |  | 40,207,966 |  | | 16,001,128 | | 2,263,584 |  | | | Total length (>= 1000 bp) | | | 318,179,120 |  | | 2,465,741[10,899] | |  |
| Agriculture | 100 | 12 | 3 |  |  | 39,304,988 |  | 35,168,588 |  | | 13,998,468 | | 1,912,370 |  | | | Total length (>= 5000 bp) | | | 0 |  | | 3,684,141[15,507] | |  |
| Forest | 0 | 0 | 1 |  |  | 84,412,940 |  | 83,793,614 |  | | 34,515,906 | | 5,173,518 |  | | | Total length (>= 10000 bp) | | | 0 |  | | 1,283,822[1,604] | |  |
| Forest | 3 | 0 | 3 |  |  | 292,104,120 |  | 287,961,054 |  | | 128,358,864 | | 18,627,910 |  | | | N50 | | | 1,379 |  | | 3,913,260[4,861] | |  |
| Forest | 3 | 3 | 3 |  |  | 278,366,980 |  | 273,348,364 |  | | 101,445,812 | | 13,755,566 |  | | | N75 | | | 1,320 |  | | 3,849,401[4,966] | |  |
| Forest | 3 | 12 | 3 |  |  | 228,912,182 |  | 226,371,794 |  | | 85,702,980 | | 10,946,086 |  | | | L50 | | | 109,184 |  | | 3,944,346[4,955] | |  |
| Forest | 3 | 90 | 3 |  |  | 180,904,266 |  | 179,534,680 |  | | 65,731,660 | | 5,696,024 |  | | | L75 | | | 168,070 |  | | 4,094,579[3,817] | |  |
| Forest | 30 | 0 | 3 |  |  | 86,835,646 |  | 86,238,746 |  | | 36,874,676 | | 5,972,088 |  | | | GC (%) | | | 55.62 |  | | 3,878,945[6,057] | |  |
| Forest | 30 | 3 | 3 |  |  | 99,361,562 |  | 98,898,134 |  | | 36,709,762 | | 6,165,458 |  | | |  | | |  |  | | 3,866,057[5,956] | |  |
| Forest | 30 | 12 | 3 |  |  | 192,501,784 |  | 191,638,622 |  | | 71,993,776 | | 12,873,468 |  | | |  | | |  |  | | 3,815,405[4,850] | |  |
| Forest | 30 | 90 | 3 |  |  | 210,294,368 |  | 209,029,498 |  | | 77,586,006 | | 6,910,054 |  | | |  | | |  |  | | 3,958,751[5,186] | |  |
| Forest | 100 | 0 | 1 |  |  | 47,878,012 |  | 47,594,792 |  | | 20,390,458 | | 3,743,320 |  | | |  | | |  |  | | 1,280,582[1,528] | |  |
| Forest | 100 | 3 | 3 |  |  | 264,423,578 |  | 262,660,438 |  | | 98,651,312 | | 18,469,138 |  | | |  | | |  |  | | 3,774,640[6,582] | |  |
| Forest | 100 | 12 | 3 |  |  | 189,276,354 |  | 188,352,616 |  | | 70,702,882 | | 11,175,118 |  | | |  | | |  |  | | 3,842,464[5,408] | |  |
| Forest | 100 | 90 | 3 |  |  | 202,050,768 |  | 200,943,026 |  | | 75,178,968 | | 7,736,922 |  | | |  | | |  |  | | 3,928,154[4,915] | |  |
|  | Total sequences: | | |  |  | 3,119,992,070 |  | 3,045,397,230 |  | | 1,182,367,738 | | 165,085,278 |  | | |  | | |  |  | | 80,830,614 | |  |
|  |  | Total contigs: | |  |  |  |  |  |  | |  | |  |  | | |  | | |  |  | | 210,239 | |  |

More information on the bioinformatic processing can be found in the Materials and Methods section.

^1^ 1.5 million sequences per samples used as input to EMIRGE assembly.

^2^ Assembly stats produced using QUAST tool.

^3^ Burrows-Wheeler Aligner (BWA) mapped sequences used as input for assembly to contigs.

^4^ Numbers in brackets refers to number of contigs.

**Supplementary Table 1 (part 2/2):** Sequence stats during bioinformatic processing

|  |  |  |  |  |  | 3. mRNA processing | | | | | | | | | |
| --- | --- | --- | --- | --- | --- | --- | --- | --- | --- | --- | --- | --- | --- | --- | --- |
| Samples | | | |  |  | 3.1. Trinity assembly^5^ | |  | 3.2. BWA mapping |  | 3.3. Normalization^6^ |  | 3.4. Rfam filtering^7^ |  | 3.5. SWORD^8^ |
| Site | Time (days) | Wood ash (t ha^-1^) | N |  |  | Assembly stats (complete contig pool) |  |  | # Sequences mapped  to contigs |  | # Sequences remaining |  | # Sequences remaining |  | # Sequences annotated to gene |
| Agriculture | 0 | 0 | 3 |  |  | # contigs | 1,050,615 |  | 1,795,078 |  | 1,361,174 |  | 870,134 |  | 9,933 |
| Agriculture | 3 | 0 | 3 |  |  | # contigs (>= 0 bp) | 1,050,615 |  | 2,864,425 |  | 2,246,922 |  | 1,460,344 |  | 14,996 |
| Agriculture | 3 | 3 | 3 |  |  | # contigs (>= 1000 bp) | 21,529 |  | 2,026,659 |  | 1,563,755 |  | 993,137 |  | 11,085 |
| Agriculture | 3 | 12 | 3 |  |  | # contigs (>= 5000 bp) | 249 |  | 1,696,811 |  | 1,283,175 |  | 737,613 |  | 12,067 |
| Agriculture | 30 | 0 | 3 |  |  | # contigs (>= 10000 bp) | 9 |  | 5,593,795 |  | 4,671,898 |  | 2,577,848 |  | 19,371 |
| Agriculture | 30 | 3 | 3 |  |  | Largest contig | 16,891 |  | 1,425,577 |  | 1,169,146 |  | 677,642 |  | 6,339 |
| Agriculture | 30 | 12 | 3 |  |  | Total length | 378,381,041 |  | 1,877,430 |  | 1,560,300 |  | 762,147 |  | 7,896 |
| Agriculture | 100 | 0 | 3 |  |  | Total length (>= 0 bp) | 378,381,041 |  | 3,997,630 |  | 3,358,975 |  | 2,094,588 |  | 18,526 |
| Agriculture | 100 | 3 | 2 |  |  | Total length (>= 1000 bp) | 34,108,217 |  | 1,526,869 |  | 1,267,251 |  | 818,865 |  | 6,047 |
| Agriculture | 100 | 12 | 3 |  |  | Total length (>= 5000 bp) | 1,765,574 |  | 1,192,600 |  | 1,002,976 |  | 571,107 |  | 5,361 |
| Forest | 0 | 0 | 1 |  |  | Total length (>= 10000 bp) | 111,009 |  | 3,802,078 |  | 2,845,062 |  | 2,134,803 |  | 25,881 |
| Forest | 3 | 0 | 3 |  |  | N50 | 340 |  | 14,057,007 |  | 10,777,632 |  | 7,783,472 |  | 77,664 |
| Forest | 3 | 3 | 3 |  |  | N75 | 266 |  | 10,034,291 |  | 7,227,095 |  | 4,982,890 |  | 75,599 |
| Forest | 3 | 12 | 3 |  |  | L50 | 343,176 |  | 7,767,931 |  | 5,470,303 |  | 3,622,207 |  | 47,584 |
| Forest | 3 | 90 | 3 |  |  | L75 | 660,965 |  | 4,252,689 |  | 2,448,852 |  | 1,454,695 |  | 184,304 |
| Forest | 30 | 0 | 3 |  |  | GC (%) | 52.75 |  | 4,455,343 |  | 3,470,187 |  | 2,373,139 |  | 20,761 |
| Forest | 30 | 3 | 3 |  |  |  |  |  | 4,596,074 |  | 3,454,745 |  | 2,051,236 |  | 20,263 |
| Forest | 30 | 12 | 3 |  |  |  |  |  | 10,103,645 |  | 8,287,001 |  | 4,848,001 |  | 40,873 |
| Forest | 30 | 90 | 3 |  |  |  |  |  | 4,972,023 |  | 3,651,673 |  | 1,723,814 |  | 91,167 |
| Forest | 100 | 0 | 1 |  |  |  |  |  | 2,930,368 |  | 2,382,064 |  | 1,771,115 |  | 10,669 |
| Forest | 100 | 3 | 3 |  |  |  |  |  | 14,144,361 |  | 11,429,426 |  | 8,367,705 |  | 60,966 |
| Forest | 100 | 12 | 3 |  |  |  |  |  | 8,418,511 |  | 6,796,579 |  | 5,103,496 |  | 125,807 |
| Forest | 100 | 90 | 3 |  |  |  |  |  | 5,634,714 |  | 4,619,276 |  | 2,395,885 |  | 73,340 |
|  | Total sequences: | | |  |  |  |  |  | 119,165,909 |  | 92,345,467 |  | 56,870,429 |  | 930,948 |
|  | Total contigs: | | |  |  |  |  |  | 1,050,615 |  | 30,635 |  | 18,161 |  | 463 |

^5^ All unaligned sequences from SortMeRNA (step 1.3) were used as input for Trinity assembly.

^6^ Normalization used: Removal of all contigs with relative expression < 1/(sequences in smallest dataset).

^7^ Contigs aligned to Rfam database to detect non-coding RNA contigs followed by removal of these contigs.

^8^ SWORD was used for aligning protein contigs to M5nr protein database.

**
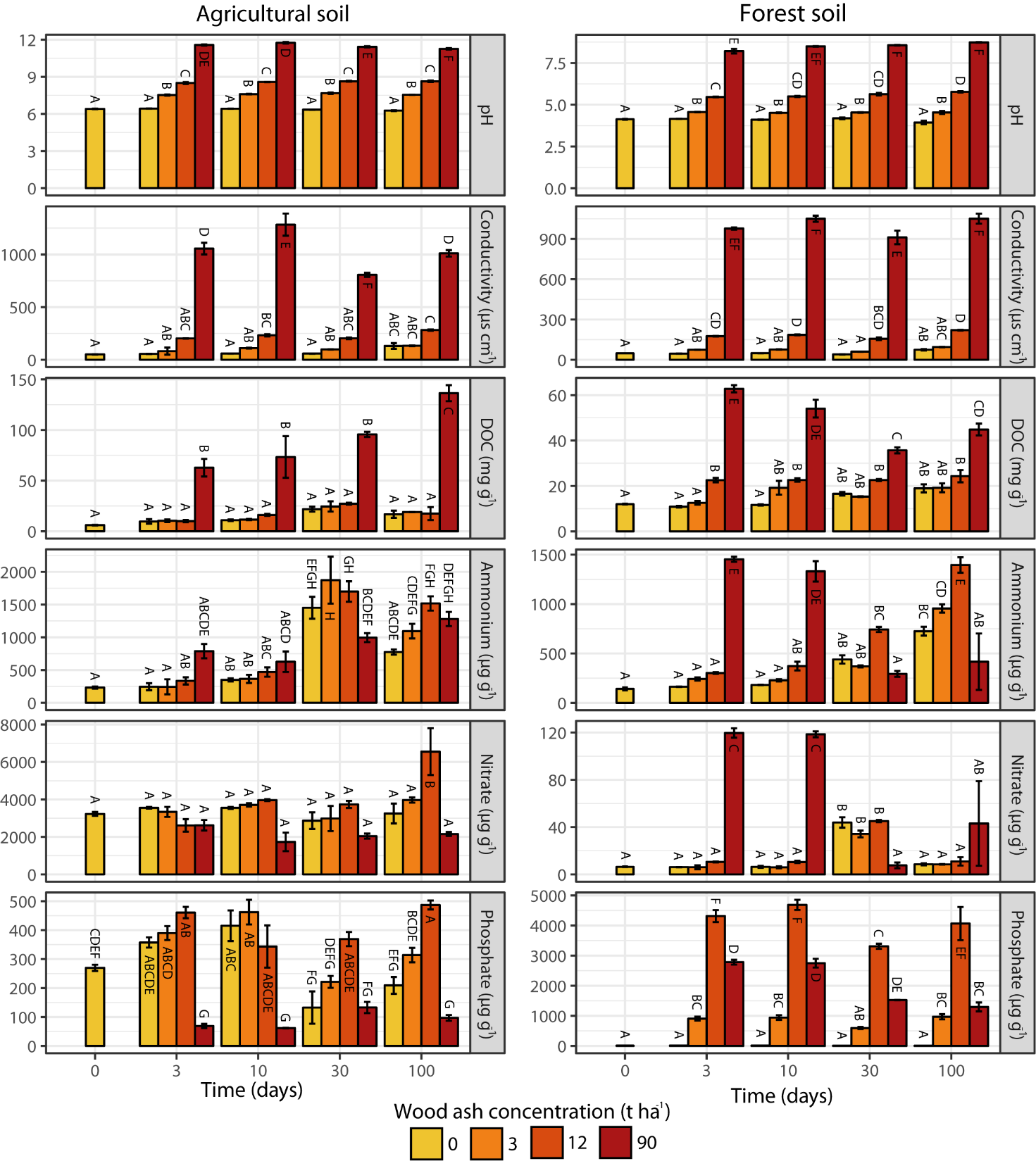
**

**Supplementary Figure 1:** Metadata results across soil types, wood ash concentrations and incubation time. Different letters denote significant (p < 0.05) difference between samples within the same plot (Tukey post-hoc pairwise comparisons). Bars represents averages of triplicates with SEM (n = 3). Bars without errorbars represents values of 1 replicate. Note different range of y-axis values between the two soils for the same metadata category.


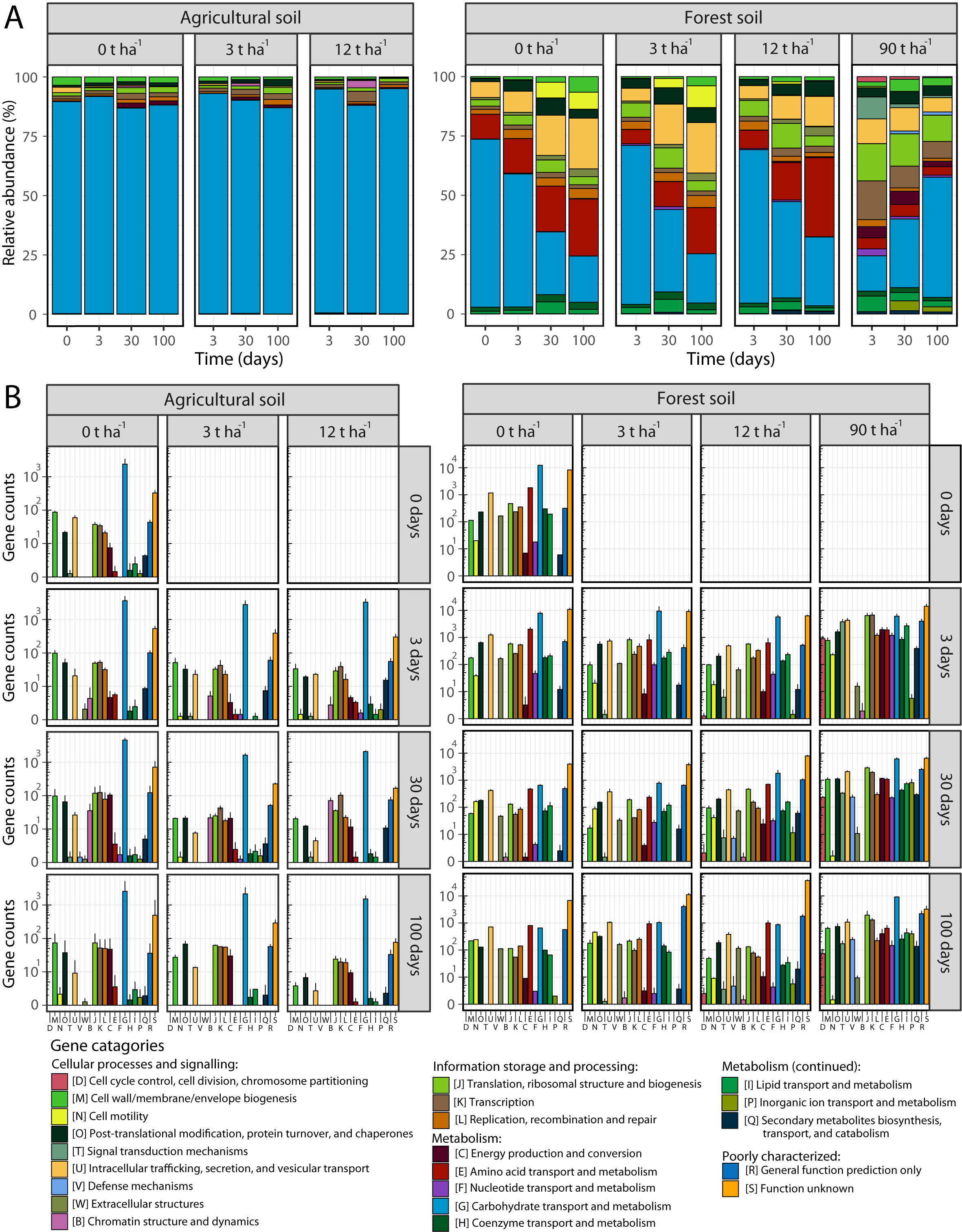


**Supplementary Figure 2:** Functional gene compositions in (A) relative abundance and (B) absolute abundance (note log10 y-axis). “Poorly characterized” genes are excluded from the relative abundance plots to increase resolution of genes with known function. Bars are averages of triplicates with SEM as errorbars (excluding agricultural soil 3 t ha^-1,^ 100 days (n=2) and forest soil 0t ha after 0 (n=1) and 100 days (n=1)).

**
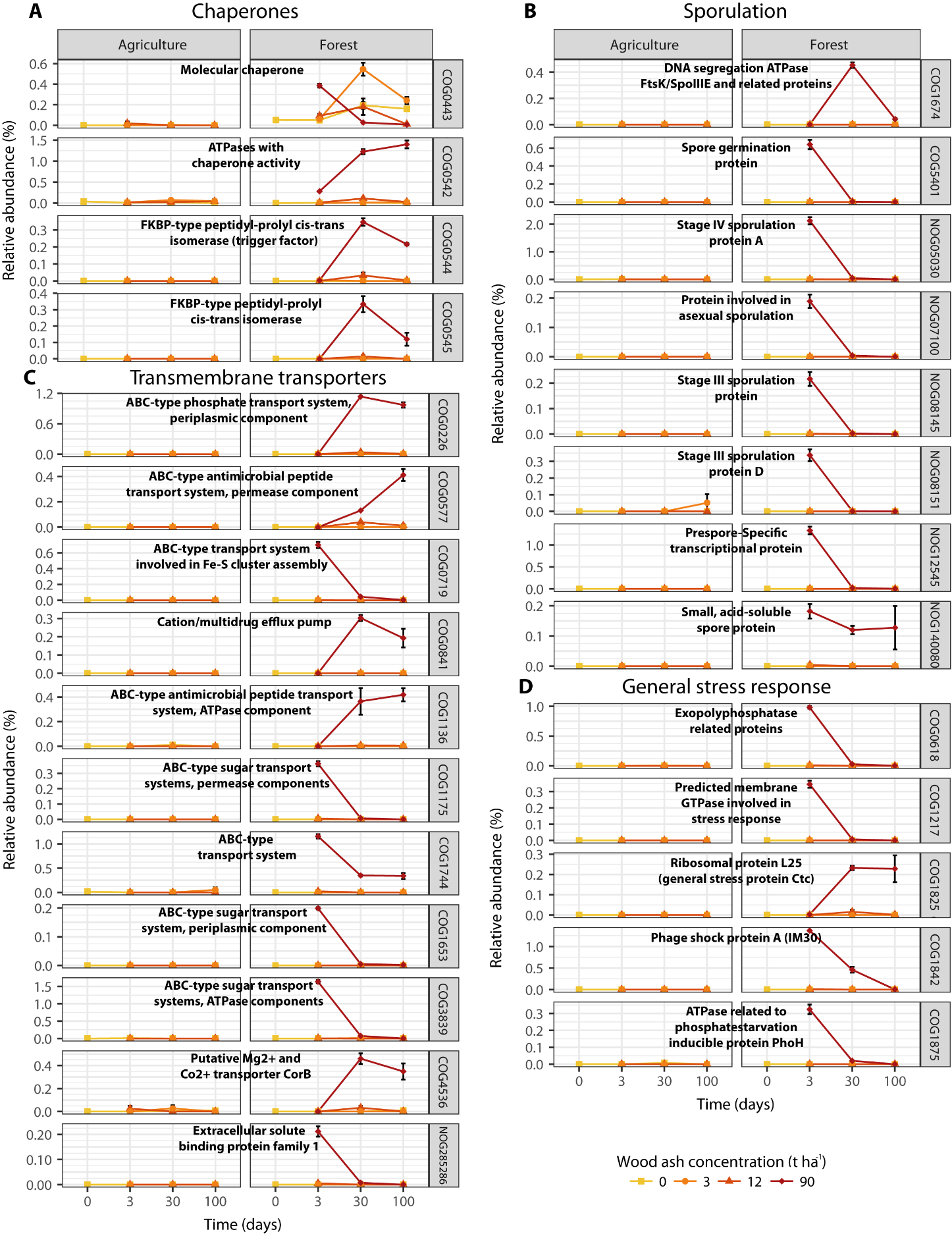
**

**Supplementary Figure 3:** Functional genes involved in (A) chaperones, (B) sporulation, (C) Transmembrane transporters and (D) general stress response. The presented functional genes (with unique COG IDs) are all differentially expressed after wood ash amendment and are presented as relative abundance of total mRNA profile. Symbols are averages of triplicates with SEM as errorbars (excluding agricultural soil 3 t ha^-1,^ 100 days (n=2) and forest soil 0t ha after 0 (n=1) and 100 days (n=1)).
